# "The Hospital" Medical Book Supplement—No. XXXIX

**Published:** 1911-02-18

**Authors:** 


### "THE HOSPITAL" MEDICAL BOOK SUPPLEMENT No. XXXIX.

CONTENTS.

| | PAGE |
|---|---|
| **The Nationalisation of Medical Practice** | 607 |
| **Therapeutics**— | |
| A Text-book of Pharmacology and Therapeutics | 608 |
| **Miscellaneous**— | |
| Practical Motherhood | 609 |
| Introduction to Practical Organic Chemistry | 609 |
| Golden Rules for Diseases of Infants and Children | 609 |
| The Saviour of the World | 609 |
| The Medical Directory for 1911 | 610 |
| Reports from the Clinical and Research Laboratories of St. George's Hospital | 610 |
| **Nursing**— | |
| The Probationer | 610 |
| Care of the Patient: A Book for Nurses | 610 |
| A Summary of Facts Regarding Malaria, Suitable for Public Instruction | 610 |

### THE NATIONALISATION OF MEDICAL PRACTICE.

A GLANCE at the columns of the chief medical journals will convince even a casual reader that the present is a time pregnant with momentous issues for the medical profession. It seems as if at last there is being awakened a keen appreciation of the social and political tendencies of the times in relation to medical practice, particularly with regard to the manner in which State interference will affect the interests of the various classes of medical practitioners. The question of sickness and invalidity insurance on a national scale is now about to enter the sphere of practical politics, and it is impossible to conceive of any scheme which will not affect enormously, not only general practice and parish work, but also the voluntary hospitals and the consultants.

### WANTED MORE SYSTEM.

That there is at present in dealing with health and disease in the community at large a vast amount of misdirected labour is obvious to any one who will take the trouble to review the workings of our overlapping and conflicting so-called systems. All misdirected labour is labour wasted, but constant association has blinded us to this want of system. Though discussions and articles in the medical press have in some measure ventilated certain aspects of this large question, it has remained for Professor Moore to come forward with a masterly and vigorous exposition in book form of the practical side of the matter.

In no uncertain voice does he proclaim the follies of our Public Health service, our methods of tinkering with disease, and the enormous waste of money and lives that is continually going on. The appearance of "The Dawn of the Health Age" (J. and A. Churchill, 3s. 6d.) at this moment is opportune, and we venture to predict a lively interest will be taken in the views set forth therein by both general practitioners and public bodies. Whatever opinions we may hold on the method of cure suggested, we cannot but welcome the outspoken way in which the failures and abuses of our present methods are expressed in this volume.

It is difficult to do justice to the author without making unduly long quotations, but we are reconciled to foregoing such by the conviction that a large circulation is in store for the book. It is indeed fitting that laymen should begin to realise those conditions under which medical practice is conducted and under which the profession itself has become so crushed and exploited. Some may be disposed to think that Professor Moore exaggerates and to congratulate themselves with the assurance that "we do not do things in that way at our hospital or in our town"; but we may admit the painful truth that we do not find any appreciable over-statement of the case. The author, in spite of his professorship, is not one who writes without experience. He knows both London and provincial hospitals, and is in a position to have a practical sympathy both with the general practitioner, who is ground between the upper and nether millstones, and the upper middle-class patient who pays doubly for the faults of our hospital systems.

### VOLUNTARYISM AND STATE AID.

In a vivid word-picture the contrast between a typical voluntary charity Hospital and a typical Poor Law Infirmary is effectively displayed. It is pointed out that "such contrasts of good and evil, sanitation and insanitation, wealth of space and overcrowding, highest medical skill and practically no medical attendance, can nowhere be found in such profusion as in the wards of our hospitals." And while, on the one hand, the very excellence of the large teaching hospitals and voluntary charities blinds our eyes to the fact that they only attend to some 15 or 20 per cent. of those requiring attention, on the other hand the Infirmaries are run on a cheese-paring attempt to save our pockets, though by the inhumanity of the process we indirectly pay tenfold, "not to mention the sacrifice of lives carried off by the infections cultured in workhouse and slumland."

We are glad to see attention drawn to another matter in connection with Poor Law Infirmaries—namely, the deplorable waste of the rich store of clinical material to be found in these institutions. Professor Moore does not mince words here, and finally suggests that by admitting students to workhouse infirmaries and thus letting in the light, not only would the whole system be exploded, but there would at once be an enormous rise in the value of the Poor Law medical appointments; in fact, the filling of these would then probably not cost the Guardians a penny. Of the awarding of appointments and of the process by which consultants are made, the author has some shrewd things to say, but where there are so many irrationalities touched upon, we cannot do more than refer to a few of the points in the book.

In a long and deductive chapter the question of tuberculosis, its spread and control, is dealt with, and incidentally it is cleverly demonstrated how the fact that a specific remedy exists for a particular disease does not necessarily have the effect of reducing the incidence of that disease in the community. As regards the control of phthisis, the point insisted upon is that it is not by a three months' sanatorium treatment of early cases that tuberculosis will be lessened in its extent, but by the segregation of the dangerous infective cases the continual crops of fresh ones would speedily diminish, and thus the accommodation at sanatoria could be gradually reduced.

It is perhaps hardly necessary to remind medical readers that Professor Moore's remedy for the shortcomings of our present methods is the institution of a National Medical Service. He seeks to do away with the heterogeneous mass of machinery and to substitute a co-ordinated and correlated plant. The main objects of the National Medical Service are to be (1) to teach the people how to live healthily, and (2) to stamp out infectious diseases with the aid of compulsory powers. Since "nearly all diseases are due either to unhealthy habits of life, or to infection from other individuals, pursuing unhealthy habits or living under unhealthy conditions, it follows that by wiping out these causes the number of diseased individuals would shrink to such dimensions that our hospitals would be well-nigh empty." It is not made clear what becomes of the doctors under these conditions, but doubtless between this present time and that Health Age there would be plenty of opportunity for compensatory re-arrangements.

The methods by which infectious diseases are notified and dealt with at present receive scathing criticism; as is pointed out, no system which requires the disease to come to the doctor, and does not allow the doctor to search for it, can be considered as efficient. We have no reason to suppose that a system which included domiciliary visits for health purposes would be resented eventually, any more than those that are now paid by sanitary inspectors, school attendance officers, and the like. The same bogey was placed very much in evidence years ago by the opponents of free education, and no doubt the cry of "the sanctity of the home" would again be raised if more militant methods of Public Health administration were to be proposed.

The lines on which a State Medical Service could be evolved require most careful consideration; the difficulties are great, and the vested interests which have to be contended with are greater, but Professor Moore is no hasty enthusiast. The discussion of many of the sub-questions is now of frequent occurrence in medical and sociological circles, and here we do no more than express the opinion that "The Dawn of the Health Age" is a book which may be profitably turned to for a realistic picture of the present state of affairs and a sane conception of some of the possibilities of the future.

### THERAPEUTICS.

A Text-book of Pharmacology and Therapeutics: or the Action of Drugs in Health and Disease. By Arthur R. Cushny, M.A., M.D., F.R.S. (London: J. and A. Churchill. 1910. Price, 15s. net.)

This revised and partly re-written edition of Professor A. R. Cushny's well-known text-book is without doubt one of the most important works on pharmacology and therapeutics in the English language. Its arrangement is such that the pharmacological and experimental observations are first adequately discussed, and then the therapeutical and practical employment of the drug is gone into in a manner that should strongly appeal to the physician on account of its conciseness and direct application to the art of medical practice. It must be admitted that in every-day medicine large numbers of drugs are used in good faith to produce certain effects for which the evidence is largely a matter of hearsay or custom. The general practitioner has often but little opportunity of revising his therapeutics; it is to be feared that the wholesale druggists are not seldom responsible for some of his advances (?) or changes. Examples of time-honoured applications that have been robbed of their reputations have been pointed out by others besides Professor Cushny, and it is well that in an authoritative text-book, such as the one under notice, mention should be made of some of these fallacies. We may allude to the effect of the local application of opium and its erroneous contra-indication in kidney disease, the virtue of belladonna plaster in staying the secretion of milk, the value of pepsin, and many other faithful superfluities. In the matter of new remedies it is fortunate that there has been a notable ebb in the flood that threatened to overwhelm us about ten years ago, and many of the minor remedies have declined considerably in use, so that the author has been able to devote more space to the important survivors. Most of these articles are valuable and concise expositions of our present knowledge: experimental and clinical evidence is now presented in instructive sequence, so that a definite and comprehensive idea of the actions of the drugs can be gained by the practitioner. Professor Cushny points out that the complex prescription is rapidly decaying, thus rendering desirable a more detailed knowledge of the individual drugs rather than the complexities of their pharmaceutical reactions, and, moreover, simplicity in prescribing will make for advance in the very direction in which progress is being made. The book is chiefly concerned with the internal effects of drugs, and the preparations mentioned are those included in the British and the United States Pharmacopœias. Important advances have been made in the study of many individual drugs, such as adrenalin and ergot, permitting a more definite statement of their action. New methods of clinical examination have thrown much light on the use of such remedies as digitalis, and the study of protozoal infections has suggested new points of view in regard to specific remedies such as arsenic and mercury. A few pages are devoted to the consideration of the ferments, secretions, and toxalbumins, but the subject of antitoxins is only briefly treated, and the question of vaccines is rightly considered outside the scope of the book. Some sixty illustrations and a bibliography at the end of each section greatly enhance the value of this handy-sized and well-printed volume.

### MISCELLANEOUS.

Practical Motherhood. By Helen Y. Campbell, L.R.C.P. and S. Edin. With illustrations. (London : Longmans, Green and Co. 1910. Pp. 535. Price 7s. 6d.)

The title of this book must be taken in its widest sense, for the author has applied, and rightly, the term to the whole range of child life from infancy to puberty. With such a wide field, it is not surprising that Dr. Helen Campbell finds over 500 pages necessary for the exposition in detail of the many subjects included. It cannot be said that brevity and conciseness of statement are strong points in this manual, yet it is perhaps hardly fair to suggest that the book would have been more useful to those for whom it is written had it been shorter. The field of child care does not only include infant feeding and nursing, but also the momentous questions connected with the development and training of the mind. The sections of the book dealing with early education form no inconsiderable proportion of the whole, and in them the writer shows freely her sympathetic temperament and her keen sense of the fitness of things in relation to the "opening mind of childhood." The philosophies of Froebel, Darwin, and the works of Stevenson, Drummond, and many other workers are frequently drawn on, while the poetic imagery of the author leavens the whole. From step to step the progress of the child is traced, and advice on the minutest points is offered to the mother; interesting treatment of such subjects as the possibilities of play and Nature study are enjoyable features. Lists of books and songs suitable for different ages are given, and, in fact, the educational value of the volume to the mother herself strikes one as being very great.

The difficult problem of sex training at puberty is by no means shirked; it is dealt with firmly and convincingly, and probably the principles expounded form the most satisfactory method of avoiding the evils commonly associated with this period. It may, we think, be questioned whether the dangers of casually acquired knowledge of sexual matters are not sometimes exaggerated, but that is no reason for neglecting the very excellent and rational schemes for imparting this knowledge, advocated and set out in this manual; healthy and clear conceptions are far more effective safeguards than virtuous ignorance. Unfortunately the qualities in the parents essential for the judicious handling of this matter are too often wanting where they are most needed. In the earlier portions of the book good advice is given on the personal hygiene of pregnancy, and a description of the salient features of labour, intended to "reassure" young mothers, is added. As the work is also written for colonial mothers, some instruction is given for those cases where help is temporarily unattainable. The subject of infant feeding is elaborated to a somewhat alarming extent; milk prescriptions cover many pages, variations and gradations almost from day to day being set out in full. The uses and abuses of patent foods and the many modifications of cow's milk are fully gone into, while a useful classification of the most widely advertised baby foods forms a valuable reference. In short, it should not be the fault of the author if this volume is consulted in vain on any detail, from the beginning of conception to the end of school life, concerning the material or moral welfare of the child. The book certainly appears to justify its title, and we may express a hope that its pages will be read with the conscientiousness with which they have undoubtedly been written.

Introduction to Practical Organic Chemistry, Including Qualitative and Quantitative Analysis and Preparations. By A. M. Kellas, B.Sc. Lond., Ph.D. Illustrated. (London : Henry Frowde and Hodder and Stoughton. 1910. Pp. 204. Price 3s. 6d. net.)

This manual is uniform with the corresponding volume on "Practical Inorganic Chemistry," which has also been recently published. The Oxford medical publications have achieved a reputation for clearness of print and general excellence of form, and these chemistry manuals are no exceptions. The author has treated the various portions of the subject in a systematic manner, and the fact that this is a book meant for use during the performance of practical work in the laboratory has always been kept in view. The range of the manual should meet the requirements of most University courses, including those of the Preliminary Scientific Syllabus (Part II.) of the London University, for which a special appendix has been written. In this appendix are to be found a description of the supplementary procedures asked at the London examination, such as Soxhlet's method of the estimation of fat and the determination of glucose by the polarimeter. There is also added a special summary of tests for the substances in Stages 1 and 2 of the Board of Education Syllabus which should be found generally useful. This book may be safely recommended to students as one from which the principles of the science may be clearly grasped.

The Saviour of the World : Vol. IV. The Bread of Life. By Charlotte M. Mason. (London : Kegan Paul, Trench, Trübner & Co., Ltd. 1910. With 12 plates. Pp. 200. Price 2s. 6d. net.)

The presentation of familiar facts and truths in a series of descriptive and contemplative verses is doubtless welcome to many. The earlier volumes of Miss Mason's exhaustive story met with a favourable reception, and this, the fourth, should maintain her reputation for reverent and graceful versification. The story of the life of Christ is here continued in this volume under the title "The Bread of Life," and is divided into four books. The writer's chief aim has been to illuminate a theme at once familiar and yet ever capable of being regarded from new points of view. The use of poetry, as the Rev. W. H. Draper remarks in the introduction, is the use of a different light—namely, that of the imagination. Twelve excellent plates, reproducing some famous religious pictures, are included in this neatly bound volume, which would form a charming gift-book.

Golden Rules for Diseases of Infants and Children. By George Carpenter, M.D. Fourth edition, revised by E. Bellingham Smith, M.D. (Bristol : John Wright and Sons, Ltd. Price 1s.)

The little books of the "Golden Rules" series have for long had an established place, not only in students' libraries but also in those of general practitioners. With a due regard for their limitations, it may be said that they are extraordinarily useful books—far more useful and much to be preferred to cram books. The volume on the "Diseases of Children" has now reached a fourth edition, and has been subjected to a thorough revision. Owing to the death of Dr. Carpenter, the book has been partly re-written and added to by Dr. Bellingham Smith, who has found it necessary to throw over the section on skin diseases, but has improved the matter in many directions. It will certainly continue to remain one of the most popular of the series.

The Medical Directory for 1911. (London: J. and A. Churchill. 1911. Price 14s. net.)

The sixty-seventh annual issue of the *Medical Directory* presents, as usual, the accustomed full and carefully edited summary of the professional life and career of the 40,642 men and women who compose the medical profession. This figure is an increase of 173 over the return for 1910; just over one-eighth are resident abroad, not counting those in the services of the Crown, who comprise about a twelfth of the total. It is interesting to notice that the decrease which started in London in 1907 still continues. The most striking new feature is the inclusion of thirty pages devoted to the principal spas and health resorts of the British Islands and the Continent, a most valuable addition on which the publishers are to be congratulated.

Reports from the Clinical and Research Laboratories of St. George's Hospital. (London: J. Bale, Sons and Danielsson. 1910. Price 5s. net.)

St. George's is one of those hospitals which have long discontinued the practice of issuing regular clinical reports; indeed, the last complete volume appeared as far back as 1879. Without discussing for the moment whether or not such reports, as still issued by some of the teaching hospitals, repay in any adequate way the time and trouble spent on their production, it will be generally agreed by everyone who reads this revived and greatly altered form that Dr. Slater has been happily inspired to undertake its publication. As he himself points out in a brief preface, medical advance and research nowadays are made so largely through the laboratory that it is advisable to focus in a volume all the work that is now being carried on in the Clinical and Research Laboratories, of which he is director. Some of the papers here collected have already appeared in print; others are published for the first time. The excretion of creatinin in various diseases is the theme of three articles: acute infective gangrene and malaria of two apiece, the laboratory diagnosis of syphilis of four. Mr. E. L. Hunt publishes a careful research into the occurrence of the *Bacillus tuberculosis* in the circulating blood of tuberculous patients, and, like most recent reseachers, arrives at conclusions entirely opposed to those of Rosenberger and Forsyth, who profess to be able to demonstrate the organism constantly. There are many other papers of bacteriological interest in the report, and the clinical aspect of disease is also by no means neglected. Dr. Slater is to be felicitated on his happy thought in thus reviving in thoroughly modern form the annual publication abandoned thirty years ago.

### NURSING.

The Probationer. By A. M. Irvine. (London: S. W. Partridge & Co., Ltd. 1910. Price 2s. 6d.)

This story of hospital life is told in a very pleasant manner, and an enjoyable evening may be spent in following the somewhat rapid career of the heroine. To those qualified to know intimately the life of a probationer some of the incidents must appear crude and far-fetched, particularly those of the opening chapters, but we may admit that many of the descriptions of ward work bear a considerable likeness to the real thing. The book may, perhaps, be read with advantage by those thinking of entering on a hospital nursing career, if due allowance be made for the romantic embellishments of the story. The story is full of humour, and the author has a certain facility in describing hospital scenes that we should look forward to his producing a more substantial hospital story in which the love element might be developed less swiftly.

Care of the Patient: a Book for Nurses. By Alfred T. Hawes, A.M., M.D. (Philadelphia: P. Blakiston, Son and Co. 1911. Price $1 net.)

Many of the procedures of practical nursing are carried out in a more or less routine manner, and probably better so, as tending to avoid the omission of some important detail. The volume under consideration is a handy summary of nursing technique pure and simple, in which the author has endeavoured to give the steps in detail of most of the procedures coming within the sphere of the nurse. The book possesses the disadvantage for English nurses in being written on American lines, but, nevertheless, it contains much that is equally applicable to home practice. The subjects dealt with cover medical, surgical, and maternity nursing, the latter being treated at considerable length. There is no attempt to go beyond the work that most nurses have to learn during their training, and, perhaps, one might add that a properly trained nurse would have had already instilled into her by continual practice nearly all the details in this book. As an aid to those learning or as a refresher to those whose hospital days are remote, it is conceivable that this manual might meet with considerable favour. In a book of so recent a date one might have expected to find some reference to the iodine method of the preparation of the skin, and it is a little startling to be told that the finding of pus at an operation necessitates the closure of the room for the purpose of its thorough fumigation with formalin. On page 96 occurs the instruction to take the temperature *by vagina* every two hours after operation; but, on the other hand, we heartily concur with the advice given on the same page, that nurses should always write "oz." and "dr." instead of employing the easily confused symbols ℥ and ʒ. Such expressions as "corrosive sponge" and "corrosive solution" are deplorable and as irritating as their literal conceptions would be. In the maternity section we find that the shaving of the parts is recommended as a routine preliminary in the preparation for labour, and that the nurse is directed to keep her hand on the fundus from the time the head is born until one hour after the placenta is delivered. An account of the administration of ether is given somewhat fully, an inclusion which we view with favour, as this is a matter which nurses should at least understand, though in this country her practical opportunities would be rare. The author is careful to admit that chloroform should never be administered by a nurse. In spite of some defects and the inclusion of certain unpractical details, we feel that there is in this book sufficient merit to make it a popular addition to some nurses' libraries.

A Summary of Facts regarding Malaria, Suitable for Public Instruction. By Major Ronald Ross. (London: John Murray. Price 2d.)

The very high opinion in which The Hospital holds Major Ross's recent book on the prevention of malaria found expression in an editorial published in these pages on November 26, 1910, p. 245. With regard to the pamphlet now published, it is only necessary to say that it is a reprint of certain passages from the major work which are especially adapted for conveying to the lay public the salient points in the ætiology, prophylaxis, and treatment of this terrible disease. Its price renders the single folio of which it consists accessible to all; the information it conveys is beyond price. An enormous circulation of it is a thing to be desired in the interests of the human race, and it may also be hoped that it may be translated into as many foreign languages as possible.

